# Music training is related to late ERP modulation and enhanced performance during Simon task but not Stroop task

**DOI:** 10.3389/fnhum.2024.1384179

**Published:** 2024-04-22

**Authors:** Miguel A. Velasquez, Jenna L. Winston, Sandeepa Sur, Kate Yurgil, Anna E. Upman, Stella R. Wroblewski, Annabelle Huddle, Paul J. Colombo

**Affiliations:** ^1^Department of Psychology, Tulane University, New Orleans, LA, United States; ^2^Department of Psychological Sciences, Loyola University New Orleans, New Orleans, LA, United States; ^3^Department of Neurology, Johns Hopkins School of Medicine, Baltimore, MD, United States; ^4^Brain Institute, Tulane University, New Orleans, LA, United States

**Keywords:** music training, EEG, N100, N450, Stroop task, Simon task, inhibitory control, ERP

## Abstract

Increasing evidence suggests that music training correlates with better performance in tasks measuring executive function components including inhibitory control, working memory and selective attention. The Stroop and Simon tasks measure responses to congruent and incongruent information reflecting cognitive conflict resolution. However, there are more reports of a music-training advantage in the Simon than the Stroop task. Reports indicate that these tasks may differ in the timing of conflict resolution: the Stroop task might involve early sensory stage conflict resolution, while the Simon task may do so at a later motor output planning stage. We hypothesize that musical experience relates to conflict resolution at the late motor output stage rather than the early sensory stage. Behavioral responses, and event-related potentials (ERP) were measured in participants with varying musical experience during these tasks. It was hypothesized that musical experience correlates with better performance in the Simon but not the Stroop task, reflected in ERP components in the later stage of motor output processing in the Simon task. Participants were classified into high- and low-music training groups based on the Goldsmith Musical Sophistication Index. Electrical brain activity was recorded while they completed visual Stroop and Simon tasks. The high-music training group outperformed the low-music training group on the Simon, but not the Stroop task. Mean amplitude difference (incongruent—congruent trials) was greater for the high-music training group at N100 for midline central (Cz) and posterior (Pz) sites in the Simon task and midline central (Cz) and frontal (Fz) sites in the Stroop task, and at N450 at Cz and Pz in the Simon task. N450 difference peaks occurred earlier in the high-music training group at Pz. Differences between the groups at N100 indicate that music training may be related to better sensory discrimination. These differences were not related to better behavioral performance. Differences in N450 responses between the groups, particularly in regions encompassing the motor and parietal cortices, suggest a role of music training in action selection during response conflict situations. Overall, this supports the hypothesis that music training selectively enhances cognitive conflict resolution during late motor output planning stages.

## 1 Introduction

The capacity for self-regulation and engagement in goal-directed behaviors constitutes essential components of executive function that contribute to success in various life domains such as academic success (Duncan et al., 2007; Borella et al., 2010; Masten et al., 2012), quality of life (Brown and Landgraf, 2010; Sanz et al., 2018) and overall mental wellbeing (Barch, 2005; Barkley, 2010; Alves et al., 2014). A growing body of evidence suggests that musical training is related to enhanced performance in several tasks used to assess components of executive function (Colombo et al., 2020) including working memory (Chan et al., 1998; Jakobson et al., 2008; Parbery-Clark et al., 2009, 2011; George and Coch, 2011; Strait et al., 2012a,b; Suárez et al., 2016), problem solving, cognitive flexibility [see Okada and Slevc (2016) for a review], and inhibitory control (Moreno and Farzan, 2015; D’Souza et al., 2018).

While music-training related cognitive benefits have been reliably demonstrated over the years, the diversity of measures used to assess executive function components across studies may contribute to divergent findings at the task-specific level. One example of this divergence is found in studies testing the relationship between music training and performance in which the Stroop or Simon tasks are used to measure cognitive conflict resolution.

The Stroop and Simon tasks share similarities as they both probe executive function by creating cognitive conflict that must be resolved in order to respond correctly. In the Stroop task, stimuli are presented with conflicting features, such as the word “red” written in blue ink. Individuals are required to respond to one feature and ignore the other, hence resolving the conflict at the stimulus stage of processing. The Simon task requires stimuli with certain features to be associated with a right- or left-hand button-press response, such as the letter “N” requiring a response from the left hand. In the Simon task, conflict resolution between task irrelevant information and response is required when the stimulus associated with one response side appears on the opposite side of the space. In this example, the letter “N” can be presented on the left side (congruent) or on the right side (incongruent). This situation requires the resolution of conflict during the response stage of processing. Evidence shows that the Stroop and Simon effects occur at different stages of cognitive processing (Simon and Berbaum, 1990; Wang et al., 2014; Scerrati et al., 2017) and, therefore, they may be subserved by different mechanisms. Scerrati et al. (2017) showed that the behavioral reaction time distribution for Stroop-like and Simon effects were different, however, this analysis does not provide insight about the differences in the timing of conflict resolution between both tasks. Examining event-related potentials (ERPs) provides a valuable method for understanding the temporal aspects of cognitive processes. Wang et al. (2014) provided evidence that during conflict processing, sensory-related ERPs occur earlier in the Stroop task than in the Simon task, showing that the resolution of conflict begins at different points in these two tasks.

Whether or not there is a music-training-related advantage in the Stroop or Simon tasks remains inconclusive. Several investigations have demonstrated a musician-related behavioral advantage for the visual Stroop, evident as a reduced Stroop effect (Travis et al., 2011; Jentzsch et al., 2014; Okada and Slevc, 2018; Strong and Mast, 2019; Chen et al., 2020) while others have shown no advantage (Zuk et al., 2014; Slevc et al., 2016; Vasuki et al., 2016; Sachs et al., 2017; Smayda, 2017). Comparisons between musicians and non-musicians on the Simon task are less frequent, but among those conducted, more show a musician advantage, characterized by a lower Simon effect (Bialystok and DePape, 2009; Amer et al., 2013; Jentzsch et al., 2014, Schroeder et al., 2016; Joret et al., 2017) than not (Slevc et al., 2016).

While it remains uncertain whether the effect sizes are larger for the Simon task than for the Stroop task among musicians, it’s notable that a higher proportion of studies demonstrate a behavioral advantage in the Simon task compared to the Stroop task. Although, there is wide variability in musicianship criteria and experience among these studies, this difference in results suggests that musical experience may have a selective, rather than global, impact on cognitive conflict resolution at specific stages of processing.

Only a few studies have examined the influence of music training on ERP components during Stroop and Simon tasks. Research indicates that musicians exhibit higher mean amplitudes than non-musicians, but the findings are specific to certain components. Jentzsch et al. (2014) reported this difference during visual Stroop and Simon tasks at early ERP components, while Chen et al. (2020) observed it during the Stroop at late components. Importantly, neither study reported differences between musical experience groups across both early and late components in Stroop and Simon tasks. In MRI studies, a greater gray matter density has been observed in musicians in the left inferior frontal gyrus (Left IFG) (Sluming et al., 2002; James et al., 2014) which may be involved in inhibition (Swick et al., 2008). Therefore, it is possible that music training is related to hemispheric differences in frontal areas that correspond to the inferior frontal gyrus.

The aim of the current project was to investigate the relationship between levels of musicianship and Stroop and Simon task performance at different processing stages. Based on previous evidence suggesting stronger musician advantage in the Simon task than the Stroop task it was hypothesized that music training is selectively related to cognitive conflict resolution at the motor-output response stage, where conflict occurs during the Simon task. This stage of conflict reflects skills practiced during musical training which involve coordination between motor responses and sensory input. In contrast music training seems less related to conflict resolution at the stimulus processing stage where conflict occurs in the Stroop task. In order to test music training-related differences in the time course of conflict resolution within the two tasks, event-related potentials (ERP) were recorded through electroencephalography (EEG). We expected a music training-related advantage on the Simon task to be accompanied by an enhanced event-related potential (ERP) response at a later, thus decisional, stage of processing. For the Stroop task, we did not expect significant ERP component differences related to music training.

## 2 Materials and methods

### 2.1 Participants

The study was comprised of 33 individuals initially. However, 10 participants were eliminated due to excessive noise in EEG signals and missing data, thereby reducing the final sample size to 23 participants (16 females and 7 males; Age M: 19.2 ± 1.5, range: 18–25 years). These participants were sourced from Sona Systems^1^ at Tulane University and received course extra credit as compensation. The study was approved by Tulane University’s Institutional Review Board.

### 2.2 Experimental procedure

Upon arrival, participants provided their written informed consent and completed the Goldsmith Musical Sophistication Index (Gold-MSI) (Müllensiefen et al., 2014). Subsequently, participants performed visual Stroop and Simon tasks in a counterbalanced order while neural activity was measured using EEG. The stimuli were presented on a computer screen placed behind a glass panel but within the participant’s view; this setup is designed to reduce electrical interference during EEG recording.

Stroop task: Participants underwent a Stroop color and word test (adapted from Stroop, 1935). The task involved presenting one of four color words (“red,” “blue,” “yellow,” “green”) in one of the four ink colors (red, blue, yellow, green). Participants were instructed to focus on the ink color, ignore the word, and select the ink color using the corresponding button on the response pad. Each participant completed a 30-trial practice block followed by five experimental blocks of 120 trials. Incongruent trials were categorized as instances where the ink color and the word did not match, accounting for 20% of trials, while the remaining 80% were congruent trials. Incongruent and congruent trials were presented in a pseudorandomized order within each block.

Simon task: The Simon task (Craft and Simon, 1970; Simon, 1990) displayed either an “H” or an “N” on either the left or right side of a middle fixation cross. Regardless of the letter’s screen position, participants were required to respond with the leftmost button on the response pad for an “H” and the rightmost button for an “N”. Each participant completed five blocks of 120 trials and an additional 15-trial practice block. The trial distribution was identical to the Stroop task, with 20% incongruent trials and 80% congruent trials.

Goldsmith Musical Sophistication Index (Gold-MSI): The questionnaire was comprised of 18 questions designed to assess musical sophistication. The self-report tool evaluates differences in musical skills in the general, non-specialist population, and gauges diverse aspects of musical sophistication, including active engagement, perceptual abilities, singing abilities and emotional responses to music. In addition, the number of years of formal training was measured as it has shown associations with performance in Simon (Slevc et al., 2016; Joret et al., 2017) and Stroop tasks (Hao et al., 2023). Based on the results of the Gold-MSI, participants were split at the median (Median = 2 years of training, Mean = 3.4 ± 2.2, Range = 0–10 years) into high- (Mean = 5.86 ± 3.5 years of training) and low- (Mean = 0.3 ± 0.4 years of training) musical training groups for subsequent analyses.

Both the Stroop and Simon tasks were conducted using E-Prime 3.0 software [Psychology Software Tools (2016), Pittsburgh, PA, USA], and the Chronos response box [Psychology Software Tools (2016), Pittsburgh, PA, USA] recorded participant responses.

### 2.3 EEG Recording and preprocessing

Participants were fitted for a standard 10–20 32-channel active electrode cap containing Ag/AgCl electrodes (Acticap, Brain Vision). The “active” electrodes contain noise subtraction circuits that significantly reduce electrical interference. A reference electrode was placed at a frontal-central midline site (FCz). Electrodes were filled with a non-toxic conductive gel in order to lower impedances, then electrodes were connected to an EEG signal amplifier and recording software (LiveAmp, Brain Vision, LLC). The impedance for each electrode was kept at or below 25 kΩ. Recordings were digitized at 500 Hz.

Electroencephalography (EEG) data preprocessing was conducted using the Brain Vision Analyzer software (BrainVision Analyzer (Version 2.2.0) (2019), Brain Products GmbH, Gilching, Germany). Trials with incorrect responses (mean incorrect Simon: 1.82; mean incorrect Stroop: 10.34) were excluded from final averages, and a band-pass filter from 0.1 to 30 Hz was applied. No specific data reduction parameters were used. Channels were referenced to the average mastoids. The data was segmented from 100 ms before the stimulus onset to 800 ms afterward, with the 100 ms pre-stimulus interval serving as the baseline for correction. Data segments were visually inspected for artifacts, and eye blink correction was performed using independent component analysis (ICA).

### 2.4 Statistical analysis

#### 2.4.1 Behavioral analysis

Mean reaction times for the Stroop and Simon tasks were obtained for each participant and congruency condition, with incorrect trials excluded. Simon and Stroop effect measures were calculated by subtracting congruent from incongruent reaction times. *T*-tests were performed on the difference between incongruent and congruent reaction times to assess Stroop and Simon effects. Analysis of variance (ANOVA) was used to examine the differences between high- and low-musical training groups in Stroop and Simon measures.

#### 2.4.2 ERP data analysis

Separate analyses of variance were used for each task and measure across musical training level and congruence condition. All ERP analyses were conducted using R Statistical Software (v4.2.1; R Core Team, 2021) and Brain Vision Analyzer software [BrainVision Analyzer (Version 2.2.0) (2019), Brain Products GmbH, Gilching, Germany].

To make comparisons between ERP components and musical experience, participants were divided into two groups [low-musical (*n* = 12) and high-musical training (*n* = 11)] based on the median years of formal music instrument training. Comparisons between the two musical training groups were made for congruent, incongruent, and difference (incongruent–congruent) waveforms.

Full assessment of ERP components’ topography involved analyzing the following five dependent measures for incongruent, congruent, and difference waveforms: mean amplitude, peak amplitude, latency, slope, and area under the curve. Analyses included electrode sites Cz, Fz, Pz and ERP components N100, P200, P300, N450. Additional analyses were conducted on sites F7 and F8 to test for hemispheric differences.

Mean amplitude: Based on the topographical distribution of the grand-averaged ERP activities, the mean amplitudes were obtained from the following time windows: N100, 90–120 ms; P200, 170–240 ms; P300, 320–380 ms; and N450, 450–530 ms.

Peak amplitudes: For each participant, peaks were obtained by using the peak detection transform function in Brain Analyzer by calculating the local maximum and minimum within the time windows defined above.

Latency: The latency was measured as the time-point after the stimulus onset when the ERP component reaches its peak amplitude.

Slope: The slope was determined by conducting a linear regression analysis. This analysis was carried out between the series of amplitudes from the point of initial deflection to the ERP peak, and the corresponding time series during this interval. The beta coefficients derived from this analysis were used as measures of the slope.

Area under the curve: Areas under the curve were calculated for each ERP component using the trapezoid rule.

## 3 Results

### 3.1 Behavioral results

Stroop and Simon effects were tested by comparing incongruent and congruent reaction times of correct trials within each task. Across all participants, incongruent trials were significantly slower than congruent trials for both Stroop (*t* = −6.47, *p* < 0.001) and Simon (*t* = −3.38, *p* < 0.001).

**FIGURE 1:**
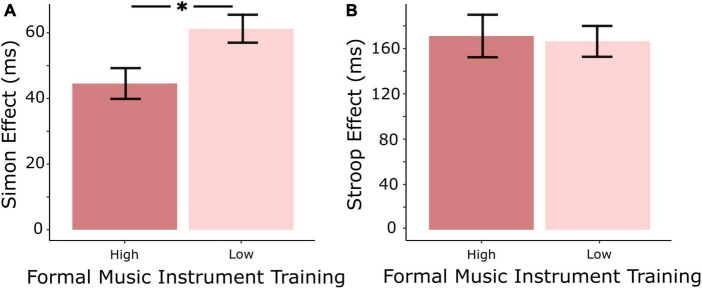
**(A)** The high formal instrument training group had a significantly smaller Simon effect (incongruent—congruent) than the low formal training group. **(B)** There was no significant difference between the two groups in the Stroop effect (incongruent—congruent). *Indicates significant difference between the means (*p* < 0.05).

The high formal instrument training group had a significantly smaller Simon effect than the low training group [*F*_(1,21)_ = 6.92, *p* = 0.014], however, there was no significant difference between the two groups in the Stroop effect [*F*_(1,21)_ = 0.04, *p* = 0.84] (see Figure 1). After Bonferroni-Holm correction for multiple comparisons, there were no significant differences in error rates between high and low training groups for either Stroop or Simon congruent or incongruent trials (see Table 1). None of the calculated Gold-MSI scales yielded a significant relationship to either Stroop or Simon behavioral performance.

**TABLE 1:** Stroop and Simon behavioral performance in low- and high- music training groups.

Simon					
	**Congruent**		**Incongruent**		
	**Reaction time**	**Error rate**	**Reaction time**	**Error rate**	**Simon effect**
Low-music training	391.66 ± 56.8	0.008 ± 0.007	452.0 ± 58.1	0.013 ± 0.0094	61.0 ± 17.7
High-music training	390.9 ± 43.2	0.004 ± 0.005	436.55 ± 64.08	0.005 ± 0.006	44.89 ± 16.98
**Stroop**					
	**Congruent**		**Incongruent**		
	**Reaction time**	**Error rate**	**Reaction time**	**Error rate**	**Stroop effect**
Low-music training	657.3 ± 82.6	0.026 ± 0.02	820.4 ± 127.4	0.05 ± 0.03	163.1 ± 58.5
High-music training	633.95 ± 60.36	0.03 ± 0.02	793.55 ± 83.53	0.08 ± 0.05	159 ± 53.96

### 3.2 ERP results

#### 3.2.1 Incongruent—Congruent waves

**FIGURE 2:**
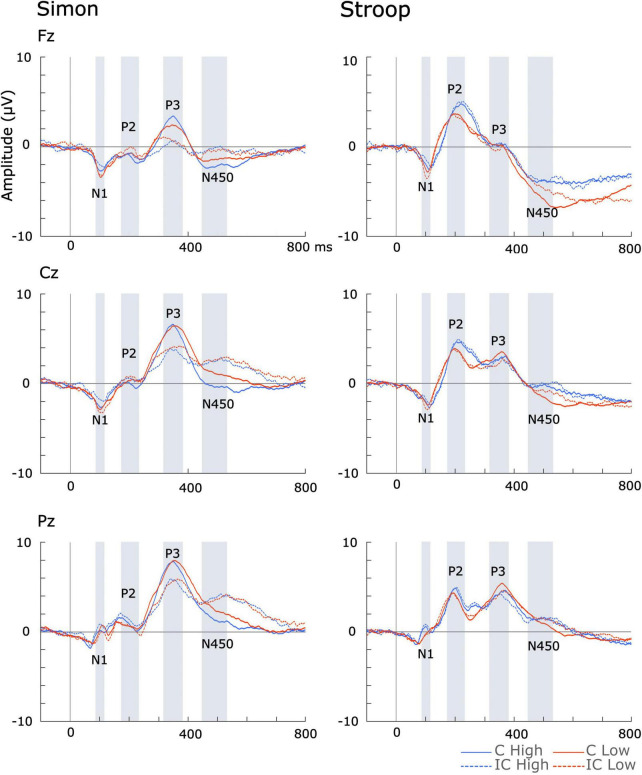
Incongruent (IC) and congruent (C) ERP waveforms across high and low formal music instrument training.

Across all measures, there were no significant differences between high- and low-musical training groups’ incongruent or congruent waves in either Stroop or Simon tasks (see Supplementary materials 1 and Figure 2).

#### 3.2.2 Difference waves

##### 3.2.2.1 Mean amplitude

The N100 mean amplitude difference for the Simon task was greater for the high music-training group than the low music-training group at Cz [*F*_(1,21)_ = 6.8, *p* = 0.017, *q* = 0.026] and Pz [*F*_(1,21)_ = 6.913, *p* = 0.016, *q* = 0.026] but not at Fz [*F*_(1,21)_ = 0.83, *p* = 0.37]. The N100 mean amplitude difference for the Stroop task was greater for the high music-training group than the low music-training group at Cz [*F*_(1,21)_ = 6.55, *p* = 0.018, *q* = 0.027] and Fz [*F*_(1,21)_ = 7.43, *p* = 0.01, *q* = 0.027] but not Pz [*F*_(1,21)_ = 2.19, *p* = 0.15].

The N450 mean amplitude for the Simon task was significantly greater for the high than low music training group at Cz [*F*_(1,21)_ = 6.55, *p* = 0.018, *q* = 0.027] and Pz [*F*_(1,21)_ = 6.54, *p* = 0.018, *q* = 0.04] but not at Fz [*F*_(1,21)_ = 2.23, *p* = 0.15]. There were no significant differences in mean amplitude at N450 during the Stroop task between the two groups.

There were no significant differences in mean amplitude at P2 or P3.

##### 3.2.2.2 Peak amplitude

**FIGURE 3:**
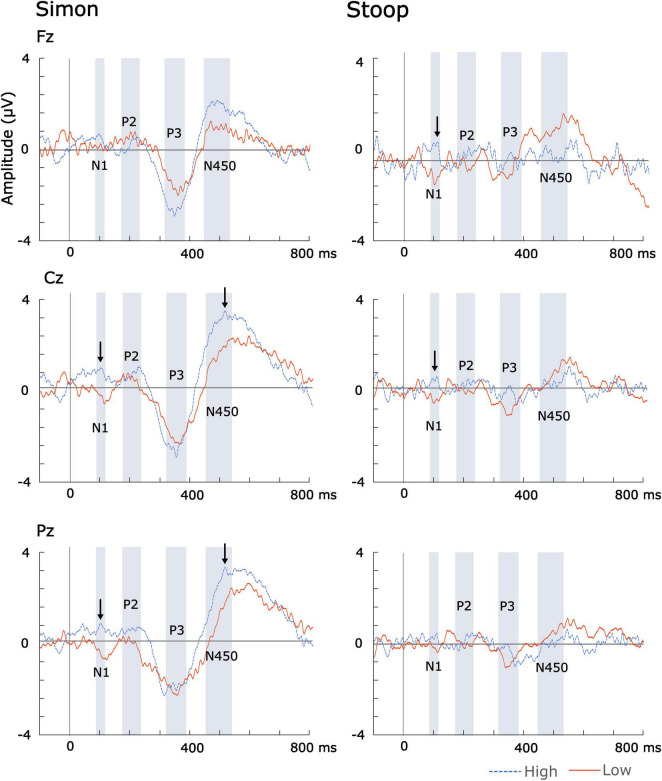
ERP difference waveforms across high and low formal music instrument training. Significant group differences are indicated by arrows.

The N100 peak amplitude difference was significantly greater in the high music training group than in the low music training group at Cz [*F*_(1,21)_ = 4.89, *p* = 0.039, *q* = 0.04] and Fz [*F*_(1,21)_ = 5.00, *p* = 0.039, *q* = 0.04] during Stroop (see Figure 3).

There were no other significant differences in peak amplitude for other ERP components or electrode sites.

##### 3.2.2.3 Peak latency, AUC and slope

The N450 peak latency for the Simon task was significantly earlier in the high music training group than the low music training group at Pz [*F*_(1,21)_ = 7.41, *p* = 0.013, *q* = 0.03].

There were no other significant differences between low and high music training groups in latency, area under the curve, or slope for any other ERP components or electrode site.

##### 3.2.2.4 Hemispheric differences

The F8 (right hemisphere) electrode site showed significantly higher peak amplitude for both P300 [*F*_(1,21)_ = 12.23, *p* < 0.001] and N450 [*F*_(1,21)_ = 9.14, *p* = 0.003] than F7 (left hemisphere) during the Simon task for all participants and across both incongruent and congruent conditions after adjusting for multiple comparisons. However, there were no significant interactions between musicianship and channel or channel and congruency condition (see Supplementary materials).

Differences in peak amplitude, mean amplitude, latency, area under the curve, and slope were tested at F7 and F8 between musicians and non-musicians across N100, P200, P300 and N450. There were no significant effects indicating a hemispheric difference between musicians and non-musicians for these measures after Bonferroni-Holm correction for multiple comparisons (see Supplementary materials).

## 4 Discussion

The aim of the present study was to investigate the association between music training and specific temporal stages of cognitive conflict resolution. It was hypothesized that music training would be selectively related to the late motor output stage of cognitive conflict resolution. To test this hypothesis, participants with various levels of music training completed Stroop and Simon tasks while EEG was recorded. The results indicated that participants with high formal music instrument training had a significantly smaller Simon effect than participants with low music-training, while there were no significant group differences in Stroop task performance. ERP analyses revealed that the high formal training group also had higher mean amplitude difference at N450 over midline central (Cz) and posterior (Pz) areas during the Simon task. N450 difference peak activity over the midline posterior region also occurred earlier in the high music training group than the low music training group during the Simon task. These differences were not observed in the Stroop task. It was also observed that N100 mean amplitude difference was significantly higher in the high music training group at midline frontal (Fz) and posterior (Pz) areas during the Simon task and over midline frontal (Fz) and central (Cz) areas during the Stroop task.

### 4.1 High formal music training is related to cognitive conflict resolution at the motor output stage

This study’s hypothesis predicted that music training selectively enhances cognitive conflict resolution at the late motor output stage. It was predicted that high music training is related to better performance and enhanced late ERP activity in the Simon, but not Stroop task. Consistent with prior research (Amer et al., 2013; Schroeder et al., 2016; Joret et al., 2017), the high formal training group had a smaller Simon effect than the low formal training group, while no significant differences were found in the Stroop task performance, suggesting a selective influence of musical training. Contrary to Amer et al.’s (2013) findings, which involved professional musicians with an average of 19.8 years of formal training, in the current study a significant difference in reaction time (RT) between the high and low training groups was not observed, although the high training group did show a numerically faster RT.

In order to test the influence of music training on the time course of cognitive conflict resolution in Stroop and Simon, the topography of early and late ERP components was studied. Wang et al. (2014) found that there was no difference in the timing of late activity, occurring between 410 and 560 ms, between Stroop and Simon-like tasks in response to incongruent stimuli. However, in the current study it was observed that the N450 difference peak during the Simon task appeared earlier in the high music training group compared to the low music training group, suggesting that music training may modulate the timing of this activity. Late ERP activity in the posterior electrode sites during Simon-like tasks may be associated with activity from the superior parietal lobule and posterior cingulate cortex (Wittfoth et al., 2006; Frühholz et al., 2011). Increased gray matter density has been observed in the posterior parietal lobe in musicians compared to non-musicians (Gaser and Schlaug, 2003). Likewise, the superior parietal lobe has been shown to be involved in sight reading (Stewart et al., 2003), a task where rapid integration of sensory inputs and motor response is necessary. The superior parietal lobe plays a crucial role in integrating various sensory information and guiding motor actions, functions that are vital for performing musicians. Matching visual patterns with motor execution is vital in musical practice and could potentially aid in resolving conflicts between sensory stimuli and motor responses like those seen in the Simon task.

In the current study high formal training was related to higher mean amplitude difference at N450 over midline central (Cz). It has been reported that N450 in central regions during cognitive conflict resolution may originate from the anterior cingulate cortex (Liotti et al., 2000; West, 2003; Badzakova-Trajkov et al., 2009; Markela-Lerenc et al., 2009), a region that has shown heightened activation in musicians during musical improvisation compared to non-musicians (Berkowitz and Ansari, 2008; de Aquino et al., 2019) and has been reported to be overall involved in conflict monitoring (Weissman et al., 2005; Botvinick, 2007; Braem et al., 2017). With connections to both motor and prefrontal cortices (Paus, 2001), the anterior cingulate cortex contributes to generating novel motor sequences (Berkowitz and Ansari, 2008). In the context of musical practice, during training musicians are required to execute new motor plans and need to consistently resolve conflicts between sensory feedback and motor responses.

Musicians have demonstrated higher N450 activity and better behavioral performance than non-musicians in fronto-central areas during visual (Chen et al., 2020) and auditory (Sharma et al., 2019) Stroop tasks. Given the greater musical experience of participants in these studies compared to the current study, it is plausible that advantages in the Stroop task are only discernible at higher levels of musicianship. Future research should explore factors that contribute to the enhanced conflict resolution observed in musicians, particularly whether different levels of musical expertise selectively improves resolution of conflicts between sensory features, as in the Stroop task, or between sensory stimuli and motor responses, as in the Simon task.

In conclusion, lower Simon effect observed in the high music training group, coupled with heightened and more rapid ERP difference wave at N450 during the Simon task is consistent with the hypothesis that music training selectively enhances cognitive conflict resolution during the later stages of motor output planning. Overall, these results indicate that the relationship between high musical training and cognitive conflict resolution is selective for resolution between stimulus and motor response conflicts but not for resolution between conflicting stimulus features.

### 4.2 Increased N100 activity in musicians may occur in response to sensory stimuli but is unrelated to attentional or inhibitory control

The N100 was detected over midline central (Cz) and posterior (Pz) areas during the Simon task, and over midline central (Cz) and frontal (Fz) areas during the Stroop task. Notably, the direction of the N100 difference waveform varied between the high and the low music training group. In the high music training group, the N100 difference waveform had a positive deflection while the low music training group displayed a negative deflection (see Figure 3). This indicates that, in the high music training group, the average N100 amplitude was smaller (more positive) during incongruent trials compared to congruent trials, while the opposite pattern was observed in the low music training group (see Figure 2). Previous studies have also reported smaller N100 amplitudes during incongruent trials compared to congruent trials in Stroop (Yu et al., 2015) and Simon tasks (Melara et al., 2008). Melara et al. (2008) further noted that larger N100 amplitudes were more likely when stimulus probability was high (at least 75% of trials), regardless of the stimulus. These results might indicate that music training may facilitate the detection of stimuli based on its probability as well as enhance the brain’s ability to differentiate between congruent and incongruent stimuli.

To our knowledge, no other studies have examined the N100 component in relation to musical experience during visual inhibitory control tasks. Putkinen et al. (2021) found that musically trained children exhibited a smaller negative N100 response to distracting novel sounds during a visual categorization task. The authors interpreted this result as evidence of a more efficient neural processing of task-irrelevant auditory stimuli in musically trained children.

The current findings raise questions about the role of the N100 component in attentional and inhibitory control, since the high music training group differed from the low training group only in the Simon task, no relationship can be established between N100 and behavioral performance in the Stroop task. This result aligns with the findings by Baumann et al. (2008), where selective attention to specific sound features did not impact N100 peak potentials in musicians, suggesting that the reported enhanced auditory-evoked responses in musicians stem from an enlarged neuronal representation for sound features rather than heightened neuronal activity due to focused attention. Overall, these results indicate that the increased N100 activity in musicians may occur in response to sensory stimuli but is unrelated to attentional or inhibitory control. The question of whether this same pattern of N100 activation spans multiple sensory modalities (such as visual and auditory) in musically trained individuals necessitates additional investigation.

### 4.3 No hemispheric differences in ERP activity between high and low musical training

In the current study no significant difference in lateralized activity was found between musicians and non-musicians. While prior research has identified left hemispheric asymmetry during auditory processing (Behroozmand et al., 2014; Black et al., 2017), to our knowledge, there is currently no evidence of hemispheric differences between musicians and non-musicians during cognitive conflict resolution tasks. Moreover, we did not observe musicianship-related differences in slope, area under the curve or peak amplitude. It is worth noting that our data displayed substantial variability in these measures, suggesting that larger sample sizes might be necessary to unveil potential differences in these aspects.

### 4.4 Individual components of musical experience are related to specific components of cognitive processing

A significant association was found between years of formal instrument training and the Simon effect, consistent with findings from prior research (Jentzsch et al., 2014; Slevc et al., 2016). However, no significant relationship was found between any of the Goldsmith Musical Sophistication Index (Gold-MSI) subscales and performance on the Stroop or Simon tasks. These outcomes suggest that the link between musical training and cognitive conflict resolution could be specific to the amount of formal instrument training, a relationship that may be weaker or non-existent when among other specific factors related to musical experience. Additionally, it is worth noting there is a large body of literature that shows that other music experience factors, not related to conflict resolution, are related to other components of cognition (Chan et al., 1998; Jakobson et al., 2008; Parbery-Clark et al., 2009, 2011; George and Coch, 2011; Strait et al., 2012a,b; Porflitt, 2014; Moreno and Farzan, 2015; Okada and Slevc, 2016, 2018; Suárez et al., 2016; D’Souza et al., 2018; Chee et al., 2022).

### 4.5 Limitations

The empirical results reported here should be considered in light of some potential limitations. The sample size used in the present study is relatively small in comparison to other studies of its kind, which may limit the generalizability of the findings. An additional limitation in this study is that the sample had a narrow range of music training. A wider range of music expertise would allow for a more fine-grained analysis of how different levels of music expertise can influence cognitive conflict resolution. Future research with larger sample size and with a wider range of musical experience could provide additional insight and would expand on these initial findings. Additionally, the present study did not manipulate musical training so conclusions about the causal relationship between music training and cognitive conflict resolution cannot be made. Finally, this study did not capture certain information about the participants’ musical training like information about participants’ primary instrument or the age of onset of musical training. These variables could also have an impact on cognitive conflict resolution.

## 5 Conclusion

Overall, the finding that higher formal instrument training was associated with both a smaller Simon effect and a larger, earlier N450 difference wave during the Simon task, provides support for the hypothesis that music training is selectively related to performance in tasks that require conflict resolution during motor output stage. The differences between the high and low music training groups at N100 indicate that music training may be related to better sensory discrimination. These differences, however, were not related to better behavioral measures of conflict resolution. Differences in N450 responses between groups with high and low levels of music training, particularly in regions encompassing the motor and parietal cortices, suggest a potential role of music training in aiding action selection during response conflict situations. These results are consistent with our hypothesis that music training selectively enhances cognitive conflict resolution during late motor output planning stages.

## Data availability statement

The raw data supporting the conclusions of this article will be made available by the authors, without undue reservation.

## Ethics statement

The studies involving humans were approved by the Tulane University Human Research Protection Office. The studies were conducted in accordance with the local legislation and institutional requirements. The participants provided their written informed consent to participate in this study.

## Author contributions

MV: Data curation, Formal analysis, Investigation, Project administration, Software, Supervision, Visualization, Writing – original draft, Writing – review & editing. JW: Conceptualization, Methodology, Project administration, Resources, Writing – review & editing. SS: Data curation, Formal analysis, Supervision, Writing – review & editing. KY: Methodology, Project administration, Resources, Supervision, Validation, Writing – review & editing. AU: Data curation, Software, Writing – review & editing. SW: Formal analysis, Writing – review & editing. AH: Data curation, Writing – review & editing. PC: Conceptualization, Funding acquisition, Methodology, Project administration, Resources, Supervision, Validation, Writing – review & editing.

## Footnotes

1
https://www.sona-systems.com

## Supplementary material

Supplementary Table 1 Congruent, incongruent and difference wave means and SD across different ERP measures during Simon and Stroop tasks.
