# Supplementary material for: Music training is related to late ERP modulation and enhanced performance during Simon task but not Stroop task
Source: Front Hum Neurosci. 2024 Apr 22;18:1384179. doi: 10.3389/fnhum.2024.1384179 (PMC11070544; doi:10.3389/fnhum.2024.1384179)
Supplement: Supplementary file 1 [file Table_2.DOCX]

| Task | Channel Pair | ERP | Measure | Music | | Channel | | Music X Channel | |
| --- | --- | --- | --- | --- | --- | --- | --- | --- | --- |
|  |  |  |  | F | p | F | p | F | p |
| Simon | F7 F8 | P300 | Peak | 1.62 | 0.21 | 0.67 | 0.42 | 0.97 | 0.33 |
| Simon | F7 F8 | N450 | Peak | 0.36 | 0.55 | 0 | 0.99 | 0.169 | 0.68 |
| Stroop | F7 F8 | P300 | Peak | 1.63 | 0.21 | 1.07 | 0.31 | 0.47 | 0.5 |
| Stroop | F7 F8 | N450 | Peak | 0.58 | 0.45 | 0.27 | 0.61 | 0.86 | 0.36 |
| Simon | F7 F8 | P300 | Latency | 0.09 | 0.77 | 1.57 | 0.22 | 3.05 | 0.09 |
| Simon | F7 F8 | N450 | Latency | 0.07 | 0.8 | 0.11 | 0.74 | 2.68 | 0.11 |
| Stroop | F7 F8 | P300 | Latency | 1.73 | 0.196 | 0.95 | 0.34 | 0.12 | 0.73 |
| Stroop | F7 F8 | N450 | Latency | 0.34 | 0.56 | 3.79 | 0.06 | 2.03 | 0.161 |
| Simon | F7 F8 | P300 | MA | 1.99 | 0.17 | 0.65 | 0.43 | 0.35 | 0.55 |
| Simon | F7 F8 | N450 | MA | 0.167 | 0.69 | 0.68 | 0.41 | 0.28 | 0.6 |
| Stroop | F7 F8 | P300 | MA | 1.94 | 0.17 | 0.001 | 0.98 | 0.001 | 0.98 |
| Stroop | F7 F8 | N450 | MA | 0.34 | 0.56 | 1.1 | 0.31 | 0.32 | 0.58 |
| Simon | F7 F8 | P300 | Slope | 1.41 | 0.24 | 0.48 | 0.49 | 0.69 | 0.41 |
| Simon | F7 F8 | N450 | Slope | 0.77 | 0.39 | 0.85 | 0.36 | 0.96 | 0.33 |
| Stroop | F7 F8 | P300 | Slope | 5.99 | **0.019, .076** | 0.002 | 0.97 | 0.27 | 0.61 |
| Stroop | F7 F8 | N450 | Slope | 3.99 | 0.052 | 2.36 | 0.13 | 4.85 | **0.03, .12** |
| Simon | F7 F8 | P300 | AUC | 5.92 | **0.02, .08** | 2.76 | 0.1 | 0.072 | 0.79 |
| Simon | F7 F8 | N450 | AUC | 0.53 | 0.47 | 0 | 0.99 | 0.139 | 0.711 |
| Stroop | F7 F8 | P300 | AUC | 0.003 | 0.96 | 0.473 | 0.5 | 0.68 | 0.42 |
| Stroop | F7 F8 | N450 | AUC | 0.16 | 0.69 | 0.003 | 0.96 | 1.36 | 0.25 |

Table 2. Hemispheric analyses in difference waveforms

| Task | Chan Pair | ERP | Measure | Mus | | Chan | | Condition | | Mus X Chan | | Mus X Condition | | Chan X Condition | | Music X Channel X Condition | |
| --- | --- | --- | --- | --- | --- | --- | --- | --- | --- | --- | --- | --- | --- | --- | --- | --- | --- |
|  |  |  |  | F | p | F | p | F | p | F | p | F | p | F | p | F | p |
| Simon | F7 F8 | P300 | Peak | 0.041 | 0.8 | 12.23 | 0.0008 | 1.755 | 0.19 | 4.35 | 0.04,.72 | 0.189 | 0.66 | 0.032 | 0.85 | 0.026 | 0.87 |
| Simon | F7 F8 | N450 | Peak | 0.62 | 0.43 | 9.14 | 0.00 | 0.00 | 0.96 | 1.10 | 0.30 | 0.17 | 0.68 | 0.01 | 0.93 | 0.04 | 0.84 |
| Stroop | F7 F8 | P300 | Peak | 0.37 | 0.55 | 0.54 | 0.47 | 0.02 | 0.90 | 1.77 | 0.19 | 0.04 | 0.84 | 0.16 | 0.69 | 0.03 | 0.87 |
| Stroop | F7 F8 | N450 | Peak | 0.28 | 0.60 | 0.22 | 0.64 | 0.03 | 0.87 | 6.91 | 0.01 | 0.03 | 0.85 | 0.04 | 0.85 | 0.03 | 0.87 |
| Simon | F7 F8 | P300 | Latency | 0.00 | 0.99 | 0.18 | 0.67 | 1.37 | 0.24 | 0.12 | 0.73 | 0.17 | 0.68 | 0.12 | 0.73 | 0.04 | 0.85 |
| Simon | F7 F8 | N450 | Latency | 0.82 | 0.37 | 0.18 | 0.67 | 1.97 | 0.16 | 0.64 | 0.43 | 0.07 | 0.80 | 1.03 | 0.31 | 0.21 | 0.64 |
| Stroop | F7 F8 | P300 | Latency | 0.22 | 0.64 | 1.48 | 0.23 | 3.75 | 0.06 | 0.01 | 0.92 | 0.26 | 0.61 | 1.07 | 0.30 | 0.46 | 0.50 |
| Stroop | F7 F8 | N450 | Latency | 0.37 | 0.54 | 0.04 | 0.84 | 0.11 | 0.74 | 0.03 | 0.86 | 1.20 | 0.28 | 3.71 | 0.06 | 0.00 | 1.00 |
| Simon | F7 F8 | P300 | MA | 0.12 | 0.73 | 10.93 | 0.00 | 4.06 | 0.05 | 1.90 | 0.17 | 0.64 | 0.43 | 0.18 | 0.67 | 0.09 | 0.76 |
| Simon | F7 F8 | N450 | MA | 1.16 | 0.28 | 6.83 | 0.01 | 0.26 | 0.61 | 0.49 | 0.49 | 0.04 | 0.84 | 0.19 | 0.66 | 0.10 | 0.75 |
| Stroop | F7 F8 | P300 | MA | 0.00 | 0.98 | 0.68 | 0.41 | 0.22 | 0.64 | 1.05 | 0.31 | 0.12 | 0.73 | 0.00 | 1.00 | 0.00 | 0.99 |
| Stroop | F7 F8 | N450 | MA | 0.49 | 0.49 | 0.27 | 0.60 | 0.02 | 0.88 | 7.16 | 0.01 | 0.01 | 0.93 | 0.04 | 0.85 | 0.02 | 0.90 |
| Simon | F7 F8 | P300 | Slope | 0.69 | 0.41 | 0.00 | 0.95 | 0.23 | 0.64 | 1.29 | 0.26 | 3.43 | 0.07 | 3.44 | 0.07 | 0.27 | 0.61 |
| Simon | F7 F8 | N450 | Slope | 5.55 | 0.02 | 0.11 | 0.74 | 10.83 | 0.00 | 0.99 | 0.32 | 0.00 | 0.96 | 0.43 | 0.52 | 0.09 | 0.76 |
| Stroop | F7 F8 | P300 | Slope | 0.00 | 0.96 | 0.89 | 0.35 | 2.30 | 0.13 | 0.00 | 1.00 | 0.13 | 0.72 | 2.05 | 0.16 | 0.01 | 0.94 |
| Stroop | F7 F8 | N450 | Slope | 0.12 | 0.73 | 0.21 | 0.65 | 3.85 | 0.05 | 1.98 | 0.16 | 0.09 | 0.76 | 0.33 | 0.57 | 2.26 | 0.14 |
| Simon | F7 F8 | P300 | AUC | 1.94 | 0.17 | 0.72 | 0.40 | 0.39 | 0.53 | 1.40 | 0.24 | 0.24 | 0.62 | 0.05 | 0.83 | 0.04 | 0.83 |
| Simon | F7 F8 | N450 | AUC | 0.15 | 0.70 | 0.43 | 0.51 | 0.07 | 0.79 | 0.71 | 0.40 | 2.63 | 0.11 | 0.04 | 0.84 | 0.02 | 0.90 |
| Stroop | F7 F8 | P300 | AUC | 1.37 | 0.25 | 0.11 | 0.74 | 0.05 | 0.83 | 0.57 | 0.45 | 0.00 | 0.96 | 0.00 | 0.97 | 0.11 | 0.74 |
| Stroop | F7 F8 | N450 | AUC | 0.41 | 0.53 | 0.09 | 0.77 | 0.07 | 0.79 | 3.00 | 0.09 | 0.08 | 0.78 | 0.01 | 0.91 | 0.06 | 0.81 |

Table 3. Hemispheric analyses in congruent and incongruent waveforms
